# Association of placenta organotin concentrations with growth and ponderal index in 110 newborn boys from Finland during the first 18 months of life: a cohort study

**DOI:** 10.1186/1476-069X-13-45

**Published:** 2014-06-05

**Authors:** Panu Rantakokko, Katharina M Main, Christine Wohlfart-Veje, Hannu Kiviranta, Riikka Airaksinen, Terttu Vartiainen, Niels E Skakkebæk, Jorma Toppari, Helena E Virtanen

**Affiliations:** 1 National Institute for Health and Welfare, Department of Environmental Health, Toxicology and Chemical Exposure Unit, Neulaniementie 4, FI-70210 Kuopio, Finland; 2 University Department of Growth and Reproduction, Rigshospitalet, Blegdamsvej 9, DK-2100 Copenhagen, Denmark; 3 Departments of Physiology and Paediatrics, University of Turku, Kiinamyllynkatu 10, FI-20520 Turku, Finland

**Keywords:** Placenta, Obesity, Weight gain, Infant, Organotin compounds, Tributyltin, Obesogen

## Abstract

**Background:**

Humans are exposed to tributyltin (TBT), previously used as an antifouling paint in ships, mainly through fish consumption. As TBT is a known obesogen, we studied the association of placenta TBT and other organotin compounds (OTCs) with ponderal index (PI) and growth during the first 18 months of life in boys.

**Methods:**

In a prospective Finnish study, 110 placenta samples were collected from mothers of boys born in 1997–1999 with (n = 55) and without (n = 55) cryptorchidism. To account for the original study design, linear regression, weighted for sampling fractions of boys with (121/55) and without (5677/55) cryptorchidism from the total cohort, was used to study the association between placenta OTCs and children’s weight, length, growth rates and PI up to 18 months of age.

**Results:**

Placenta TBT concentrations were above the limit of quantification (LOQ) in 99% of the samples. However, monobutyltin (MBT), dibutyltin (DBT) and triphenyltin (TPhT) concentrations were below LOQ in 90%, 35% and 57% of samples, respectively. Placenta TBT was positively associated (p = 0.024) with weight gain during the first three months of life, but no other significant associations were observed for weight or length gain. Also, no significant associations between placenta OTC concentrations and child length, weight or PI at any time point were found.

**Conclusions:**

We observed a trend towards higher weight gain from birth to 3 months of age with increasing placenta TBT concentration. These results should be interpreted with caution because obesogenic effects in animal experiments were seen after in-utero TBT exposures to doses that were orders of magnitude higher. Also the number of study subjects included in this study was limited.

## Background

The organotin compounds (OTCs) tributyltin (TBT) and triphenyltin (TPhT) have strong biocidal activity and have been used e.g. in antifouling paints in boats and ships. TPhT has also been used as an agricultural pesticide [1]. The detrimental effects following antifouling use of TBT, and TPhT are of most environmental concern and have been extensively reviewed by Graceli et al. [2]. In brief, TBT is considered to be a chronic contaminant and very low concentrations in water are known have e.g. the following effects: levels > 1 ng/l limit cell division in phytoplankton and zooplankton reproduction; levels > 2 ng/l are responsible for shell calcification anomalies in the oyster *Crassostrea gigas*; levels > 20 ng/l impair bivalve mollusc reproduction by impairing the reproductive function and can induce imposex, intersex and the formation of ovo-testis. Additionally, OTs may accumulate in birds and sea mammals and can limit growth rates and photosynthesis in algae. Due to the detrimental environmental effects their use in antifouling [3] and agricultural applications [4] has been banned in Europe. Also, the scientific panel of the European Food Safety Authority (EFSA) has established a group Tolerable Daily Intake (TDI) of 250 ng/kg body weight for the sum of dibutyltin (DBT), TBT, TPhT, and dioctyltin (DOT) based on the immunotoxicity of these compounds [5]. For humans, fish is considered to be the main source of TBT and TPhT exposure [6,7]. However, PVC plastics, e.g. vinyl blinds, vinyl sidings and wallpaper, may contain large amounts of OTCs and could be a significant source of butyltin and octyltin exposure for children through direct dermal contact and dust ingestion and inhalation [8,9].

The current epidemic of overweight has sparked the discussion on the role of environmental contaminants on this development. The first systematic review on the possible contribution of low level chemical exposure to the obesity epidemic was made by Baillie-Hamilton [10] and the “obesogen” concept was launched in a commentary of this study [11]. Especially the possibility that prenatal exposure to obesogenic chemicals may permanently program those exposed to greater propensity to obesity later in life has gained considerable interest. For humans tobacco smoke is an established prenatal obesogen [12-14]. Animal experiments and epidemiological studies have listed other suspected obesogens such as diethylstilbestrol (DES), bisphenol A (BPA), phytoestrogens, organochlorine pesticides, polychlorinated biphenyls (PCBs), phthalates and perfluorinated alkyl acids (PFAAs) [15-17]. For example, a number of studies have found an association between prenatal organochlorine (OC) compound exposure and increased BMI in infancy [18], childhood [19,20] and adulthood [21].

To date, TBT is the best studied environmental obesogen. *In vitro*, TBT enhances adipogenesis, triglyceride storage and expression of adipogenic marker genes [22-24] , causes increases in adipogenic capacity and reduction of the osteogenic capacity of adipose-derived stromal stem cells (ADSCs) [25]. Also, *in utero* exposure to TBT causes lipid accumulation into various adipose depots at the expense of bone [26] and similar effects have been observed even at transgenerational level [27]. Peroxisome proliferator-activated receptor gamma (PPAR-γ) has a central role in TBT-induced adipocyte differentiation [24].

Despite good *in vitro* and *in vivo* evidence of the obesogenic effects of TBT, there are no human epidemiological studies on the topic. This study aimed at evaluating the possible impact of prenatal exposure to OTCs (monobutyltin (MBT), dibutyltin (DBT), TBT, TPhT) on the ponderal index (PI) and growth during the first 18 months of life among Finnish boys.

## Methods

The study was conducted according to the Helsinki II declaration after informed oral and written consent of the parents. The ethical committee of Turku University Hospital (Finland: 7/1996, 6/2001) approved the study.

### Study population and placenta samples

Placenta samples were obtained from a Finnish prospective, longitudinal cohort study performed in 1997–1999 at Turku University Hospital, Turku, Finland. The primary aim of this study was to establish contemporary prevalence rates for cryptorchidism and hypospadias, and at evaluating risk factors by means of a questionnaire and biological samples (blood, placenta, and breast milk). Recruitment strategy, inclusion criteria and clinical examination of the children have been described in previous publications [28-30]. Placenta samples were collected from mothers of healthy boys (n = 55) and mothers of boys with cryptorchidism (n = 55) at birth in a nested case–control design. The matching criteria were: parity, maternal smoking (yes/no), diabetes (yes/no), gestational age (±7 days) and date of birth (±14 days).

### Chemical and anthropometric measurements

The analyses used to estimate exposure were prospectively planned to include persistent and non-persistent chemicals in breast milk and placenta (EU grant QLK4-CT-2001-00269), most of which have been previously reported, including OTCs from placentas but not from breast milk [29,31-38].

To analyse OTCs from placenta samples, perdeuterated OTCs used as internal standards were added to 0.25 g of dried placenta homogenate. Homogenates were digested with tetramethyl ammonium hydroxide and methanol. Samples were then buffered to pH4 with sodium acetate-acetic acid and OTCs were ethylated with sodiumtetraethylborate. Ethylated OTCs were extracted to hexane and purified with alumina column. Purified extracts were concentrated and subjected to gas chromatography/mass spectrometry (GC/MS) analysis (Agilent 6890 GC/Waters Autospec Ultima high-resolution MS). Limits of quantification (LOQs) were 0.1 ng/g fresh weight (fw) for MBT and DBT, and 0.02 ng/g fw for TBT and TPhT.

Weight and length was obtained for 110 boys at birth, for 107 at 3 months and for 91 at 18 months. Ponderal index (PI) was calculated as kg/m^3^. Weight and length gain (kg/week, cm/week) were calculated from birth to 3 months and from 3 to 18 months.

### Statistics

Background characteristics of mothers and boys were calculated as previously described [33,34,38] The concentrations of each OTC compound and the sum of OTCs were grouped into two or three categories, depending on the distribution of concentrations of each OTC. Concentrations below the LOQ were treated as one-half of the respective LOQ in the calculations [24]. Means of weight, length and PI in each OTC category were also calculated.

Because growth rates among healthy and cryptorchid boys may differ, complex samples general linear model weighted for sampling fractions of boys with (121/55) and without (5677/55) cryptorchidism from the total cohort was used to study the association between placenta OTC concentrations and different anthropometric parameters. It has been shown that weighting for sampling fractions can be used for the analysis of case–control data for additional outcomes [39]. Impact of categorised placenta OTC concentrations on PI, weight and length at birth, 3 months and 18 months, and on weight gain (kg/week) and length gain (cm/week) between 0–3 months and 3–18 months were explored using weighted general linear model. Firstly, an analysis across OTC categories without covariates was performed to test crude associations. Secondly, an analysis across OTC categories, adjusted for maternal age at birth, maternal pregnancy body mass index, diabetes (yes/no) and smoking (yes/no) during pregnancy, and infant weight for gestational age, was performed. Weight for gestational age was calculated using national standards [40]. Most covariates were selected based on their known association with weight development in infancy and childhood. The threshold for statistical significance was set at p < 0.05. Reference groups for OTC concentrations were <0.1 ng/g fw for DBT and TBT, <0.02 ng/g fw for TPhT, and <0.25 ng/g fw for the sum of OTCs.

Information on the duration of total and/or partial breast feeding was available for 80 of 110 study subjects. A breast feeding index was constructed from these data for the periods 0–3 months and 3–18 months and included as a covariate in an additional weighted general linear model.

Dioxin and PCB levels have previously been analysed from the placenta samples of the current study [38]. Because the sum of dioxin and PCB toxic equivalences (total-TEQ) in breast milk has been associated with increased early length and weight gain [41], and because human intake of both OTCs, dioxins, and PCBs is accounted by the same source, i.e. fish [6,42] possible confounding was assessed by including log-transformed total TEQ sum as a covariate in a separate weighted general linear model. Also Sprearman rank correlation between total-TEQ and TBT was calculated.

Finally, to study the association between categorised placenta OTC concentrations and anthropometric parameters only among the healthy boys (n = 55), analysis of variance adjusted for maternal age at birth, maternal pregnancy body mass index and smoking (yes/no) during pregnancy, and infant weight for gestational age, was performed. There were no diabetics among mothers of healthy boys, and it was thus not included as covariate.

All statistical tests were performed with IBM SPSS Statistics 21, Armonk, New York, USA.

## Results

### Background characteristics and concentrations of OTCs in placentas

Population characteristics are given in Table 1 and OTC concentration in placentas in Table 2. Concentrations of OTCs were below LOQ in a large proportion of samples, especially those of MBT (90%), TPhT (57%) and DBT (35%).

**Table 1:** **Population characteristics (N, medians, 2.5**^**th **^**and 97.5**^**th **^**percentiles) for mothers and boys**

N	110
Maternal age (years)	28.2 (21.0-40.0)
Maternal pregnancy BMI (kg/m^2^)	22.8 (17.9-34.7)
Diabetes (yes, No)	9, 100
Smoking (yes, No)	17, 93
Parity	
1	60
2	38
≥3	12
Gestational age (days)	280 (256, 295)
WGA (%)^a^	−0.90 (−22.5, 22.6)
Prematurity (N)^b^	4
Weight at birth (kg)	3.59 (2.72, 4.52)
Weight at 3 months (kg)	6.48 (5.27, 7.97)
Weight at 18 months (kg)	12.0 (9.91, 14.0)
Length at birth (cm)	51.0 (47.8, 55.0)
Length at 3 months (cm)	62.3 (57.4, 65.1)
Length at 18 months (cm)	84.0 (79.7, 88.7)
PI at birth (kg/m^3^)	26.5 (21.9, 32.0)
PI at 3 months (kg/m^3^)	27.1 (22.5, 32.9)
PI at 18 months (kg/m^3^)	20.1 (16.8, 23.3)

^a^WGA = weight for gestational age was calculated using national standards as percent deviation from the expected mean (Pihkala et al. [40]), −22% being equivalent to -2SDs.

^b^Prematurity = < 259 days of gestation.

**Table 2:** **Percentage of samples < LOQ and concentrations (ng/g fw) of organotin compounds in placentas**^**a**^

MBT% < LOQ	90
Mean	0.06
Median	0.05
2.5^th^–97.5^th^ percentiles	0.05–0.18
DBT% < LOQ	35
Mean	0.14
Median	0.12
2.5^th^–97.5^th^ percentiles	0.05–0.36
TBT% < LOQ	1.0
Mean	0.32
Median	0.18
2.5^th^–97.5^th^ percentiles	0.03–1.2
TPhT% < LOQ	57
Mean	0.05
Median	0.01
2.5^th^–97.5^th^ percentiles	0.01–0.31
Sum of OTCs	
Mean	0.56
Median	0.39
2.5^th^–97.5^th^ percentiles	0.14–1.8

^**a**^In the calculation of mean, median and percentiles, LOQ/2 was used for concentrations less than the LOQ.

### OTCs and weight and length gain from 0 to 3 months and from 3 to 18 months

Placenta TBT had a significant positive association (p = 0.024) with weight gain from birth to three months when comparing the third category to the first category, and similar associations of borderline statistical significance were observed for DBT and the sum of OTCs (Table 3). No associations were observed for weight gain between 3 and 18 months. TBT had a significant negative association (p = 0.042) with length gain between 3 to 18 months, but this was not consistent across TBT categories and no other associations with length gain were found (Table 3).

**Table 3:** **Associations between placenta OTCs and weight/length gain across OTC categories in weighted linear regression**^**a**^

**Organotin compound**	**Conc. (ng/g fw)**	**Weight gain 0–3 months**				**Weight gain 3–18 months**			
		**N**	**Weight gain (kg/week)**	**β (95% CI)**	**p**	**N**	**Weight gain (kg/week)**	**β (95% CI)**	**p**
DBT	<0.10	31	0.220	ref		27	0.087	ref	
	0.10–0.14	30	0.222	0.016 (−0.011, 0.044)	0.23	26	0.082	−0.007 (−0.020, 0.006)	0.29
	≥0.15	41	0.230	0.021 (0.000, 0.043)	0.054	33	0.079	−0.009 (−0.020, 0.002)	0.11
TBT	<0.10	31	0.224	ref		26	0.086	ref	
	0.10–0.4	39	0.217	0.005 (−0.018, 0.029)	0.64	34	0.084	−0.007 (−0.022, 0.008)	0.36
	>0.4	32	0.235	0.024 (0.003, 0.044)	0.024	26	0.078	−0.011 (−0.024, 0.003)	0.12
TPhT	<0.02	60	0.227	ref		49	0.080	ref	
	≥0.02	42	0.220	0.005 (−0.016, 0.027)	0.62	37	0.085	0.002 (−0.008, 0.011)	0.74
Sum of OTCs	<0.25	26	0.220	ref		22	0.083	ref	
	0.25–0.50	31	0.228	0.025 (−0.002, 0.052)	0.065	27	0.086	<0.001 (−0.016, 0.016)	1.00
	>0.50	45	0.225	0.018 (−0.001, 0.037)	0.062	37	0.080	−0.008 (−0.021, 0.005)	0.21
**Organotin compound**	**Conc. (ng/g fw)**	**Length gain 0–3 monhts**				**Length gain 3–18 monhts**			
		**N**	**Length gain (cm/week)**	**β (95% CI)**	**p**	**N**	**Length gain (cm/week)**	**β (95% CI)**	**p**
DBT	<0.10	32	0.83	ref		28	0.34	ref	
	0.10–0.14	30	0.83	0.054 (−0.038, 0.146)	0.25	26	0.33	−0.014 (−0.039, 0.010)	0.25
	≥0.15	41	0.83	0.047 (−0.021, 0.114)	0.18	33	0.33	−0.008 (−0.027, 0.011)	0.42
TBT	<0.10	32	0.82	ref		27	0.34	ref	
	0.10–0.4	39	0.83	−0.015 (−0.085, 0.055)	0.67	34	0.33	−0.025 (−0.048, −0.001)	0.042
	>0.4	32	0.84	0.024 (−0.038, 0.085)	0.45	26	0.34	−0.014 (−0.036, 0.009)	0.22
TPhT	<0.02	60	0.83	ref		49	0.34	ref	
	≥0.02	43	0.83	−0.005 (−0.061, 0.051)	0.86	38	0.34	−0.006 (−0.025, 0.013)	0.54
Sum of OTCs	<0.25	27	0.84	ref		23	0.34	ref	
	0.25–0.50	31	0.82	0.012 (−0.084, 0.108)	0.81	27	0.34	−0.012 (−0.042, 0.018)	0.41
	>0.50	45	0.83	0.018 (−0.039, 0.076)	0.53	37	0.33	−0.012 (−0.035, 0.010)	0.29

^a^Adjusted for maternal age at birth, maternal pregnancy body mass index, diabetes (yes/no) and smoking (yes/no) during pregnancy, and infant weight for gestational age.

When only healthy boys were included, association of TBT with weight gain when comparing the third category to first category was only borderline significant (p = 0.086). Similar weakening of associations with weight gain when comparing the third category to first category were observed also for DBT (p = 0.093) and sum of OTCs (0.132).

### OTCs and infant PI at birth, 3 months and 18 months

In the unadjusted analysis no significant associations between placenta OTC concentrations and child PI at any age were found (data not shown). In the adjusted model, a statistically significant negative association between DBT and PI at birth was found, however, not systematically across all DBT categories. At three and 18 months these associations were still negative, but did not reach statistical significance (Table 4). No associations were found between placenta TBT and PI except for a borderline significant negative association at birth that was not systematic across TBT categories (Table 4). Analysis with healthy boys only did not materially change these results (not shown).

**Table 4:** **Associations between categorical OTC concentrations and PI from linear regression analysis weighted for sampling fractions**^**a**^

**Organotin compound**	**Conc (ng/g fw)**	**PI at birth**				**PI at 3 months**				**PI at 18 monhts**			
		**N**	**mean PI (kg/m**^**3**^**)**	**β (95% CI)**	**p**	**N**	**mean PI (kg/m**^**3**^**)**	**β (95% CI)**	**p**	**N**	**mean PI (kg/m**^**3**^**)**	**β (95% CI)**	**p**
DBT	<0.10	33	26.8	ref		31	27.5	ref		28	20.1	ref	
	0.10–0.14	30	26.0	−1.46 (−2.56, −0.35)	0.010		26.7	−1.35 (−2.77, 0.08)	0.063		20.0	−0.79 (−2.36, 0.79)	0.32
	≥0.15	42	27.4	−0.45 (−1.55, 0.66)	0.43	30 41	28.2	−0.10 (−1.15, 1.13)	0.99	26 33	20.0	−0.93 (−2.04, 0.18)	0.098
TBT	<0.10	33	26.5	ref		31	27.4	ref		27	20.0	ref	
	0.10–0.39	40	26.6	−1.36 (−2.90, 0.18)	0.084	39	26.9	−0.28 (−1.79, 1.24)	0.72		20.2	0.46 (−0.97, 1.90)	0.52
	≥0.40	32	27.5	−0.26 (−1.63, 1.12)	0.71	32	28.5	0.85 (−0.20, 1.90)	0.11	34 26	19.9	−0.38 (−1.33, 0.57)	0.43
TPhT	<0.02	61	27.0	ref		60	27.9	ref		49	20.1	ref	
	≥0.02	44	26.6	−0.75 (−1.56, 0.06)	0.068	42	27.0	−0.24 (−1.20, 0.73)	0.63	38	20.1	0.21 (−0.67, 1.09)	0.63
Sum of OTCs	<0.25	27	27.1	ref		26	27.3	ref		23	19.8	ref	
	0.25–0.49	32	26.0	−1.23 (−2.69, 0.24)	0.10	31	27.2	0.57 (−1.03, 2.16)	0.48		20.3	1.33 (−0.31, 2.97)	0.11
	≥0.50	46	27.3	−0.53 (−1.79, 0.74)	0.41	45	27.9	0.42 (−0.59, 1.43)	0.41	27 37	20.0	−0.33 (−1.27, 0.60)	0.48

^a^Adjusted for maternal age at birth, maternal pregnancy body mass index, diabetes (yes/no) and smoking (yes/no) during pregnancy, and infant weight for gestational age.

Inclusion of breast feeding index or total TEQ as covariates of regression analysis had little effect on the results (results not shown). TBT and total TEQ had weak negative Spearman rank correlation even though it was of borderline significance (r = −0.174, p = 0.070).

No significant associations were found between OTCs and child weight, weight for gestational age or length at any time point (data not shown).

## Discussion

To our knowledge, this is the first human study on the associations between OTC exposure and infant growth. The main finding of this study was that placenta TBT is associated with increased weight gain during first three 3 months of life. No statistically significant associations were observed for other OTCs or weight gain between 3 and 18 months.

Increased weight gain in early life may have long-term consequences due to the well-described association with increased risk of adult obesity [43,44], cardiovascular disease [45] and non insulin-dependent diabetes mellitus [46]. No association between PI and OTC exposure was found. PI is traditionally used in infancy as measure of body proportions in parallel to the use of BMI in older children. However, it was recently shown that neither PI nor BMI may be a very sensitive measure of true body fat mass [47].

We have previously described that prenatal exposure to a mixture of modern pesticides was associated with smaller birth weight and weight for gestational age, but larger increase in BMI Z-score from birth to school age. However, as in the current study, absolute measures such as body weight, length or BMI at school age showed less clear associations [41]. In addition, the sum of dioxins and PCB toxic equivalences in breast milk was associated with lower fat percentage, lower weight and length at birth, and increased early length and weight gain Z-score, i.e. rapid catch up growth. We speculated that the catch up growth associated with dioxin-like chemicals may partly be explained by a compensatory biological mechanism following Ah-driven intrauterine growth restriction [48]. However, adjustment for total TEQ did not change the results of regression analysis in our study and also the Spearman rank correlation between placenta TBT and total TEQ was weak. Thus, dioxin and PCB exposure measured as chemical levels in the placentas did not explain the associations found in this study.

Prenatal human exposure assessment through measurement of OTCs in placenta is in our opinion a highly valid approach. Gooke et al. has has performed an extensive study with Sprague–Dawley rats on the organotin speciation and tissue distribution in dams, fetuses and neonates following oral administration of TBT. On gestational day (GD) 20 in the highest exposure group (10 mg/kg bw/d), the concentrations of TBT and DBT were the highest in dams’ brain, liver and spleen (1500–1800 ng/g and 400–1900 ng/g), but were also high in the placenta (650 ng/g and 300 ng/g). Meanwhile, the levels of TBT and DBT in liver and brain of fetuses were about half of those in dams. On GD20, levels of TBT and DBT in placenta were 5-fold higher than the levels in maternal blood and 10-fold higher than in milk on post-natal day 6 [49]. Measurement of OTCs from breast milk samples of Japanese women [50] and whole blood samples of Finnish fishermen [51], both high consumers of fish, support the tissue distribution results from rat experiments. Both human matrices contained mainly non-detectable levels of TBT and barely detectable level of DBT (milk) and TPhT (blood). Thus, placenta tissue may be ideal to reflect true foetal exposure. However, as in urine, where the concentration of TBT metabolites decreased rapidly during the first days after voluntary ingestion of OTCs [52,53], OTCs may be rather quickly eliminated also from placenta. This may present a problem for intake estimation if maternal intake of OTCs varies a lot during pregnancy. Generally, body distribution of charged OTCs appears to be different from that of classical neutral POPs that are preferentially stored in lipid rich tissues including milk. This behaviour may explain why adjustment for breast feeding did not have any impact on the results.

From the mechanistic point of view, TBT is described as a high affinity agonist of PPAR-γ and retinoid X receptor (RXR), two important nuclear receptors during adipocyte differentiation [26]. An antagonist study has confirmed the importance of PPAR-γ in the TBT-induced adipocyte differentiation [24]. Also a recent study on the molecular mechanisms of TBT obesogenicity using microarray analysis in the 3 T3-L1 *in vitro* system revealed enrichment of Gene Ontology terms involved in energy and fat metabolism, and pathway analysis unveiled PPAR signaling as the most significantly enriched pathway [54]. Based on our results, the observed associations between OTC concentrations in placenta and infant weight gain, if real, was mainly due to TBT as DBT is a debutylation product of TBT [34]. In addition, the obesogenic potential of TBT through RXRα/PPARα is much stronger than that of DBT [26]. TPhT may also contribute as it has similar RXRα/PPARα affinity as TBT [23,26], but its concentration in our placenta samples was at least an order of magnitude less than that of TBT.

Estimated average dietary intake for the sum of four OTCs specified by European Food Safety Authority (DBT, TBT, TPhT and dioctyltin (DOT)) in Finland is about 3 ng/kg body weight (bw) per day [6,55]. The Tolerable Daily Intake (TDI) of 250 ng/kg bw for OTCs based on their immunotoxicity [5], is 80 times higher. In the worst case scenario of high consumption of contaminated fish the intake for women increases to 120 ng/kg body weight per day [55]. There are reasons to believe that the maternal intake of OTCs in the current study was quite high. Firstly, consumption of local fish that was heavily contaminated with OTCs during the time of sample collection [56] may have resulted in a high intake. Secondly, in another Finnish study TBT concentrations in placenta samples collected in 2004–2005 from the inland city of Kuopio were much lower, all less than the LOQ of 0.20 ng/g fw in that study [57].

The relationship between PI, used as metrics of adiposity in the current study, and OTC exposure can be complicated. In experiments with C57BL/6 J mice daily prenatal TBT exposure of 50–500 μg/kg by intraperitoneal injection from embryonic day 12 until the day before delivery significantly increased adipose mass in TBT-treated males by 20% over controls. However, growth curves for TBT-treated pups showed a slight trend for lower total body weight that was statistically not significant. TBT may thus increase body adiposity without overtly increasing total body weight [26]. Using the same mice strain a single prenatal TBT exposure of 100 μg/kg by gavage on embryonic day 16.5 led to a 2-fold increase in the total lipid accumulation (from 20% to 40%) compared with controls [25]. Maternal exposure in these animal experiments thus appears to be 1000 times higher than that of mothers in our study.

This study has several limitations. Firstly, the number of subjects was rather small increasing the risk of chance finding. Secondly, the cohort used was originally planned for the study of congenital malformations, not for the outcomes studied here. This limitation was accounted for by using a weighted general linear model approach [39]. Regarding these limitations, we note that the significant association between placenta TBT and weight gain turned into borderline significant when only healthy boys were included in the analysis. Thirdly, we used PI as a measure of infant adiposity in lack of a better biomarker. Animal and *in vitro* experiments refer to the importance of specific adiposity measurements, but unfortunately this was not available at the time the cohort was investigated.

## Conclusions

We observed that placenta TBT concentration was associated with increased weight gain during the first three months of life of newborn boys, suggesting a possible obesogenic effect. No clear associations were observed between placenta OTCs and infant PI during the first 18 months of life. The results should be interpreted with caution, because the number of study subjects included in this study was limited. The study should be repeated with a larger number of subjects, more specific biomarkers of adiposity and longer follow up time.

## Abbreviations

Bw: Body weight; BMI: Body mass index; CI: 95% Confidence interval; CV: Co-efficient of variation; DOT: Dioctyltin; EFSA: European food safety authority; fw: Fresh weight; LOQ: Limit of quantification; MBT, DBT, TBT: Mono-, di- and tributyltin; MPhT, DPhT, TPhT: Mono-, di- and triphenyltin; PI: Ponderal index; OR: Odds ratio; OTCs: Organotin compounds; WGA: Weight for gestational age; TDI: Tolerable daily intake.

## Competing interests

The authors have no competing financial, personal or professional interests.

## Authors’ contributions

PR was responsible for the chemical analysis of OTCs, performed the statistical tests and the manuscript writing. KMM, NES and JT designed the Danish-Finnish cryptorchidism study, supervised its conduct, and contributed to the interpretation of data and manuscript writing. HEV conducted the study in Finland and both contributed to the data interpretation and manuscript writing. CWV, HK and RA were involved in the data analysis and interpretation of the data. TV planned and included the analysis of OTCs for the larger cryptorchidism study. All authors read and approved the final manuscript.
